# Impact of biodegradation on the barrier performance of polymer-modified bentonite

**DOI:** 10.1371/journal.pone.0357812

**Published:** 2026-09-30

**Authors:** Natascha Kallerhoff, Eva Heinz, Wolfgang Lieske, Wiebke Baille, Marc Wichern, Torsten Wichtmann

**Affiliations:** 1 Chair of Soil Mechanics, Foundation Engineering and Environmental Geotechnics, Ruhr-University Bochum, Bochum, Germany; 2 Urban Water Management and Environmental Engineering, Ruhr-University Bochum, Bochum, Germany; 3 Geotechnical Engineering, Georg Agricola University of Applied Sciences, Bochum, Germany; University of Central Florida, UNITED STATES OF AMERICA

## Abstract

Polymer modification shows potential for improving the performance of clays in geotechnical applications. This includes its use as a filler in clay liners under challenging geotechnical and environmental conditions (e.g., exposure to aggressive leachates and intensive drying), as well as applications in soil stabilisation and as supporting fluids for mechanised tunneling and diaphragm wall construction. However, due to the organic nature of most commonly used polymers, they may serve as a substrate for microbial metabolism. The limited understanding of polymer biodegradation and its influence on bentonite behaviour raises concerns regarding the long-term stability of polymer-enhanced clays. This study investigates potential biological degradation of cationic (PAA^+^) and anionic (PAA^-^) polyacrylamide-modified bentonite under saturated conditions. Bentonite MX80 was modified via solution intercalation and subjected to 32 weeks of permeation testing. The outflow was analysed chemically and microbiologically. Following permeameter testing, the hydraulic conductivity, rheological behaviour and microbial community composition of the soil material were determined, and initial and post-test microbial communities were compared. Based on the analysis a conceptual degradation model was developed and discussed. Polymer degradation was found to depend on the presence and persistence of specific polymer-degrading microorganisms, with Pseudomonas species identified as key contributors. Although PAA^+^ modified MX80 exhibited higher microbial degradation activity, the long-term performance of polymer-modified bentonite was governed primarily by the charge-dependent polymer-clay binding mechanism rather than by the extent of polymer loss. Surface-bound PAA^+^ showed greater robustness than bridging-dominated PAA^-^ systems, as confirmed by hydraulic and rheological analyses.

## Introduction

Clays and clay-based materials are widely used in geotechnical and environmental engineering due to their low hydraulic conductivity and favorable sealing properties. They are applied in a broad range of applications, including hydraulic barriers, soil stabilisation measures, and as functional components in supporting fluids for mechanised tunneling and diaphragm wall construction. Their performance is largely governed by the interaction between the clay minerals and pore water, particularly the ability of smectitic clays to hydrate and retain water [1–3]. However, environmental and mechanical boundary conditions can significantly impair the functionality of clayey materials [4]. Changes in pore water chemistry, such as elevated salinity [5,6] or extreme pH values [7], may reduce water-binding capacity and lead to increased hydraulic conductivity. Desiccation and cyclic wetting–drying further promote shrinkage, cracking, and loss of integrity [3], while the inherently low shear strength of hydrated clays increases their susceptibility to mechanical failure [8,9]. To address these limitations, water-soluble linear polymers have gained significant attention in geotechnical applications due to their ability to modify soil properties, including mechanical strength [10], swelling stability [11,12], hydraulic conductivity [13], resistance to cracks [14] and chemical interactions [15]. Thus, polymer modification is considered as a potential agent to optimise clay performance in challenging geotechnical and environmental conditions.

## Background

### Polymer-clay-interaction

The binding mechanisms between active clay minerals, such as smectite, and polymers are influenced by several factors, including the chemical composition, charge density, molar mass of the polymer, and the mixing method [16,17]. Cationic (positively charged) polymers exhibit the strongest interactions with clay minerals due to ion-ion attraction, as the positively charged functional groups of the polymer chains adsorb irreversibly onto the negatively charged basal clay surface and partly replace exchangeable cations [18–20]. Due to the high density of adsorption points and the strong attachment of the polymer chains to the mineral surface, the binding mechanism can be described as a surface-bound system, in which the polymers are firmly anchored to the clay surface through electrostatic attraction. According to [16], this can yield a reduction of the diffuse double layers of the clay minerals, thereby promoting the formation of aggregated clay fabrics (Fig 1a). By contrast, anionic (negatively charged) polymers are predominantly adsorbed through cation bridging, whereby the polymer chains interact with cations that have been adsorbed onto the negatively charged clay surface [21]. Due to electrostatic repulsion between the polymer and the clay surface, only a limited number of adsorption points are available [18]. Intra-chain electrostatic repulsion causes the polymer chains to adopt an extended conformation, enabling them to link adjacent clay particles. Consequently, the overall interaction mechanism can be described as a bridging-dominated system, leading to the formation of flocculated clay fabrics (Fig 1b, [16]). However, several studies have demonstrated that polymer binding is not limited to surface adsorption. Instead, ionic polymer chains can penetrate into the interlayer space of highly expandable Na-smectites [22,23]. This intercalation mechanism can be activated using the so-called solution intercalation mixing method. In this approach, clay and polymers are dispersed and mixed in solution, allowing the clay interlayers to expand due to swelling and thereby increasing the likelihood of polymer penetration. The intercalation mechanism is governed by the polymer charge and its molar mass [18,22,24]. Thus, under identical mixing conditions, different intercalation efficiencies may be present.

**Fig 1:**
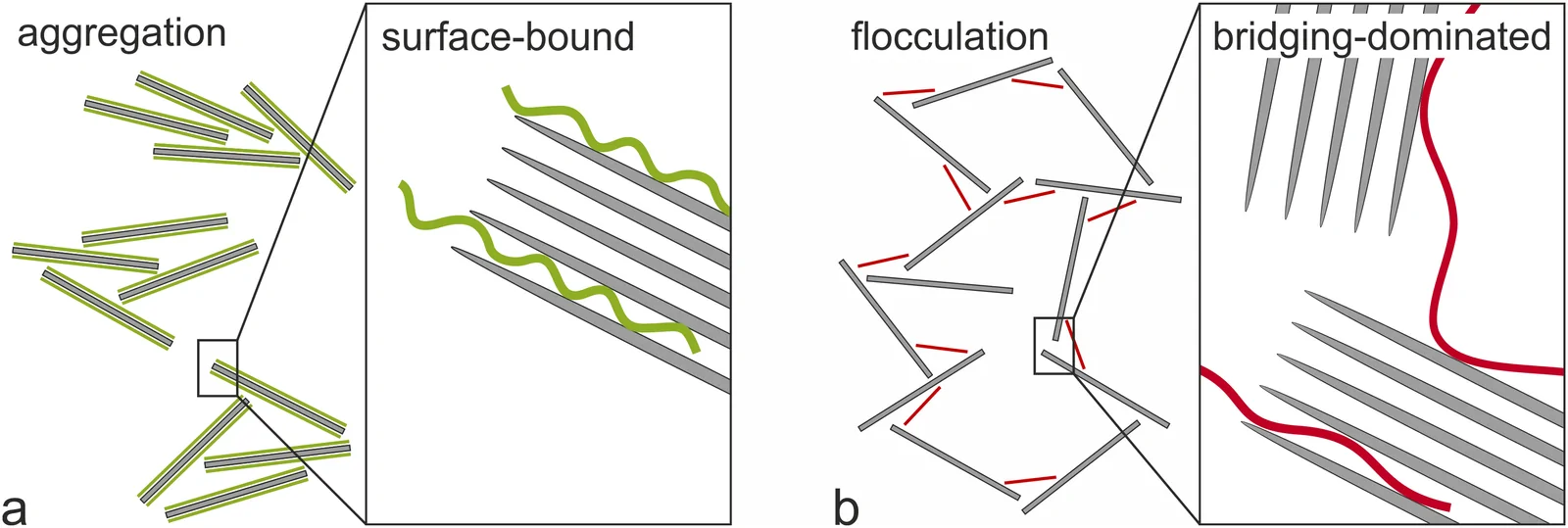
Microscale fabric of polymer-modified bentonite (modified after [16]). a: Clay-cationic polymer-composite. b: Clay-anionic polymer-composite.

### Degradation of polymer-modified clay

Polymers may undergo degradation during their service life, leading to progressive alterations in their properties, such as changes in molar mass and polymer composition. In soils, mechanical and chemical stresses, as well as biological activity, are potential drivers of degradation. The latter refers to microbiological activity by bacteria or fungi, which leads to the decomposition of polymer chains. Relevant microorganisms are naturally existing in soils, surface water and groundwater [25]. In long-term applications, biodegradation-related changes in polymer properties may compromise the functional performance of polymer-modified clays. Biodegradation of polyacrylamide occurs under both aerobic and anaerobic conditions [26], yet knowledge about the responsible microorganisms (bacteria and fungi) remains limited. Moreover, polymer degradation may be strongly influenced by the presence of smectite, as the activity of water in the vicinity of charged surfaces is reduced and may therefore inhibit biological activity [11]. Despite the relevance of this topic for the long-term stability of geotechnical barrier systems, systematic studies investigating polymer biodegradation within bentonite remain scarce. This highlights the need for an interdisciplinary investigation integrating microbiology, polymer chemistry and soil science to address polymer biodegradation in bentonite adequately. A comprehensive understanding of these degradation pathways is nevertheless essential for predicting material performance over extended timescales and ensuring the safe application of polymer-modified bentonites in environmental barrier systems.

This study investigates the chemical, microbiological, hydraulic and rheological evolution of polymer-treated bentonite under saturated conditions. Specifically, the work addresses the following research aims:

1. Identification of polymer-degrading microorganisms and temporal analysis of the developing soil microbiome.
2. Determination of degradation stages based on chemical and microbiological indicators, including nitrogen mass balance considerations.
3. Evaluation of methodological approaches suitable for quantifying polymer degradation in soils.
4. Development and analysis of a conceptual degradation model integrating chemical, microbiological, and rheological evidence.

## Materials and methods

### Soil and polymer

The commercially available bentonite MX80, sourced from Wyoming, USA contains 75% montmorillonite, 15% quartz and 5 – 8% feldspar. In the current study, the material was supplied in powdered form. The dominant absorbed cation is Na^+^ and a CEC of 85 cmol(+)/kg is reached [20]. With a liquid limit of $w_{L}$  =  588% and a plasticity index of PI  =  37%, the MX80 bentonite is classified as highly plastic clay (CH) (ASTM D2487-17). Thus, it shows pronounced colloidal and binding properties. The specific gravity was determined using the pycnometer method according to ASTM D854-23, with kerosene used as the recommended solvent [27], yielding a value of G_s_  =  2.60.

The synthetic polymers used in this study are commercially available products supplied by SNF (Andrezieux, France). Both materials are copolymers composed of two principal monomeric components and exhibit an ionicity of 40 mol%. The cationic polymer FO 4490 (PAA^+^) consists of acrylamide (prop-2-enamide) and methylated dimethylaminoethyl acrylate, whereas the anionic polymer AN 945 (PAA^-^) is composed of acrylamide (prop-2-enamide) and acrylic acid (prop-2-enoic acid) (Fig 2). Upon contact with water, PAA^+^ becomes positively charged, whereas PAA^-^ develops a negative charge. This behaviour arises from the protonation of the tertiary amine functionalities in FO 4490, yielding positively charged quaternary ammonium groups, while the acidic groups in AN 945 dissociate, releasing protons and forming negatively charged carboxylate groups. Based on the molar ionicity, the mean molar mass of the repeat units can be determined from the corresponding monomer fractions. In its hydrated state, the cationic polymer (PAA^+^) has an average repeat-unit molar mass of $M_{{PAA}^{+}}=105.93\;g/mol=0.60\cdot 71.08\;g/mol+0.40\cdot 158.22\;g/mol$, while the anionic polymer (PAA^-^) exhibits a lower average repeat-unit molar mass of $M_{PAA^{-}}=71.47\;g/mol=0.60\cdot 71.08\;g/mol+0.40\cdot 72.06\;g/mol$, reflecting the substantially lower molecular mass of the anionic monomer. The molar mass of PAA^+^ lies in the range of $7\;\text{--}\;9\cdot 10^{6}$ Da, whereas PAA^-^ exhibits higher molar masses of $12\;\text{--}\;14\cdot 10^{6}$ Da. Both polymers were included in the experimental program to assess whether their distinct interaction mechanisms with the negatively charged clay surface influence the effectiveness of polymer-clay interaction.

**Fig 2:**
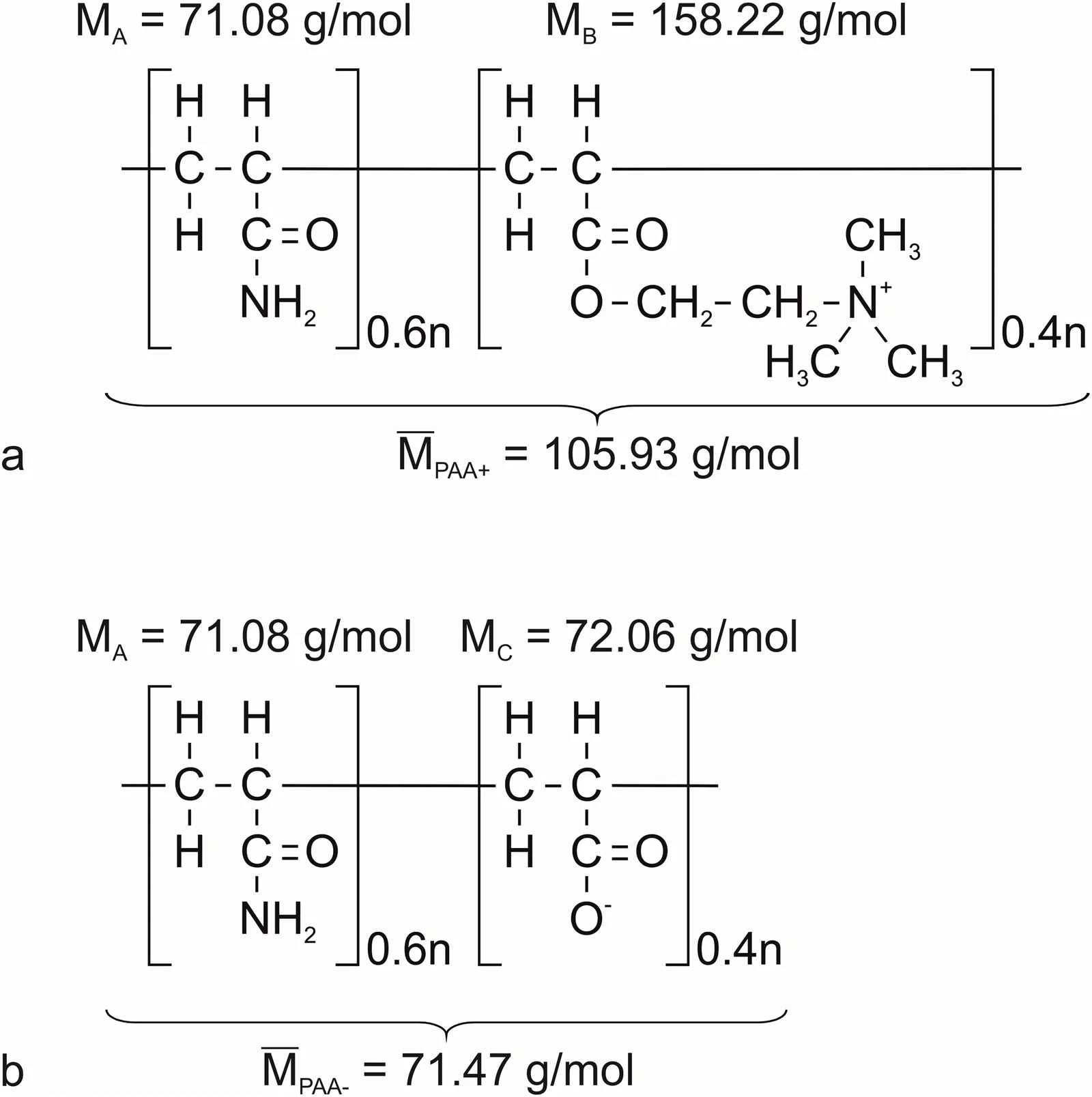
Chemical structures of the polymers, including the molar masses of the individual monomers and the average repeat-unit molar masses. a: Cationic polymer PAA^+^. b: Anionic polymer PAA^-^.

### Identification of polymer-degrading micro-organisms and method of bacterial growth curves

In order to test for the presence of polymer-degrading microorganisms in soil, bentonite MX80 was subjected to microbiological analysis. The enrichment and adaptation of microorganisms were performed according to the methodology described by [28]. From the enrichment cultures, four pure cultures of polymer-degrading microorganisms were successfully isolated. Three of the isolates were identified by 16S rRNA amplicon sequencing as members of the genus Pseudomonas (class Gammaproteobacteria), whereas the remaining isolate could not be identified.

Growth curves were generated for one of the pure Pseudomonas cultures. Therefore, the corresponding polymer was added to the basic medium (BM, composition as specified in [28]), followed by inoculation with 500 μL of a glycerol-preserved pure culture (50 % v/v). Initial growth was monitored immediately by measuring optical density (OD) and determining colony forming units (CFU). Additional samples were collected at regular intervals up to 406 h for the polymer PAA^+^ and up to 236 h for the polymer PAA^-^ for subsequent analyses.

### Preparation of polymer-modified samples and experimental test setup

Polymer-modified bentonite was prepared using the so-called ‘solution intercalation method’ [29]. The aim was to achieve a pronounced polymer–clay interaction so that polymer-modified smectite could be analysed, rather than focusing on the degradation of concentrated polymer gel entrapped within the clay pore space [12]. Therefore, the as-is bentonite MX80 was suspended in autoclaved water at a concentration of 42 g/L to ensure the homogeneous dispersion of the clay particles. Simultaneously, a separate polymer solution with a concentration of 4 g/L autoclaved water was prepared. This concentration is based on the as-supplied polymer mass, including its intrinsic hygroscopic water content of 10.5% for PAA^+^ and 20.1% for PAA^-^. Once the soil suspension and polymer solution were homogeneously mixed, they were combined and stirred continuously for an additional three hours. This ensured sufficient contact time between the polymer chains and the clay minerals, thereby promoting effective interaction between the two components. Polymer penetration into the clay interlayer space was confirmed by X-ray diffraction (XRD) measurements, as documented in [30]. The observed changes in basal spacing indicate polymer intercalation rather than solely surface adsorption. After the mixing process, the composite material was centrifuged (20 min at 4700 rpm) to remove excess liquid. The paste from the centrifuge was installed into DKS-permeameters under slurry conditions ($\sim 1.1w_{L}$) [11] and subjected to a vertical stress of 10 kPa (Fig 3). Filter stones placed above and below the sample ensure complete saturation of the material, as autoclaved water is pumped into the filter stones after installation of the initial material. After two weeks of contact with the built-in material, the water in the filter stones was pumped out and replaced with fresh autoclaved water. Exactly 50 ml of outflow was collected from each DKS permeameter every two weeks and subjected to chemical analysis (pH, electrical conductivity (EC), Ammonium NH_4_-N, filtered total nitrogen N_total,fil_) and microbiological analysis (CFU, 16S rRNA amplicon sequencing). After 32 weeks of outflow collection, the built-in materials were removed from the DKS-permeameters. Since duplicate tests were performed per condition, one dismantled sample was subjected to further microbiological analysis to characterise its microbiome using 16S rRNA amplicon sequencing. Hydraulic tests, as well as a rheological characterisation were performed on the other dismantled sample (Fig 4).

**Fig 3:**
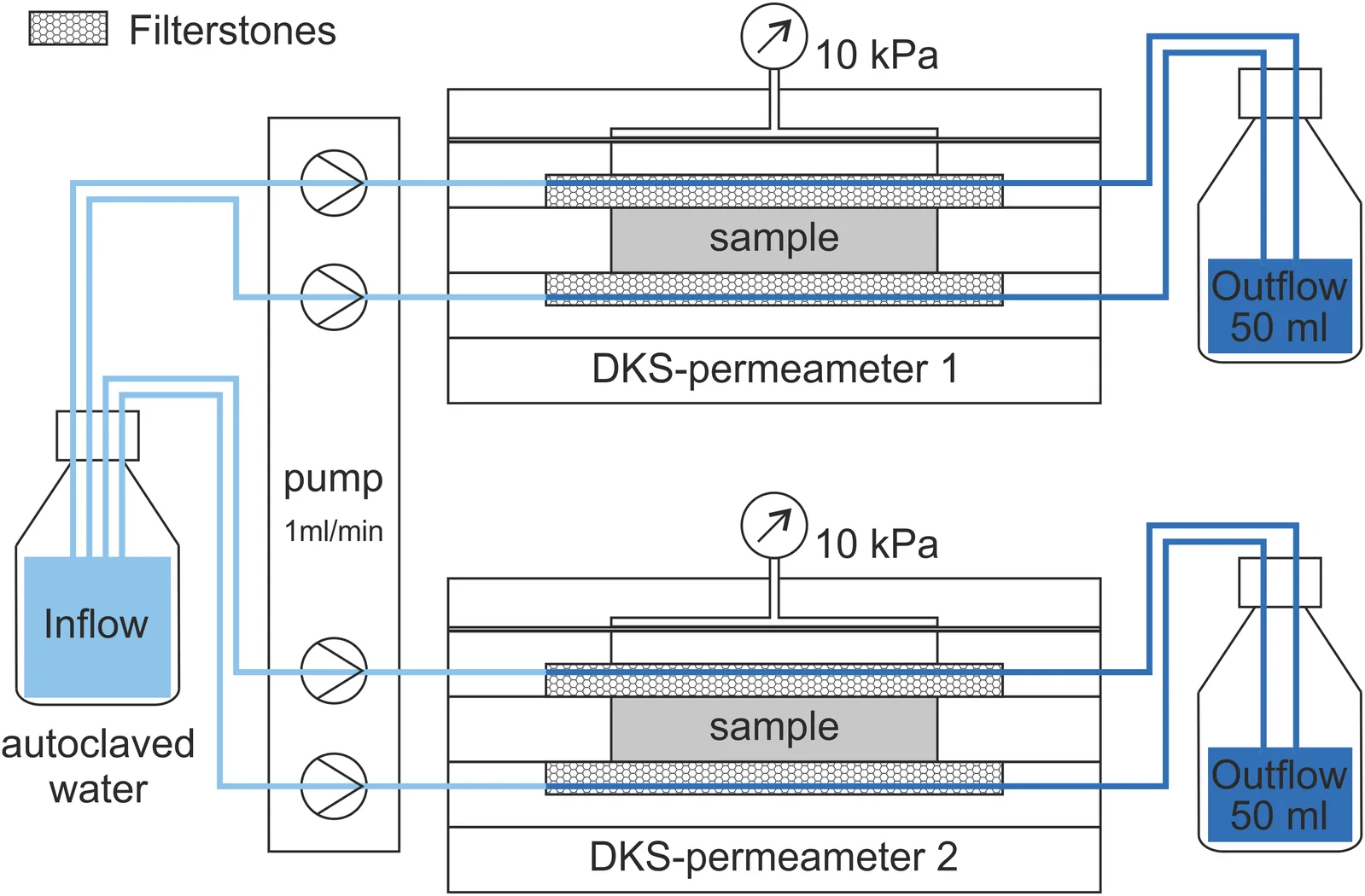
Experimental setup of the DKS-permeameter.

**Fig 4:**
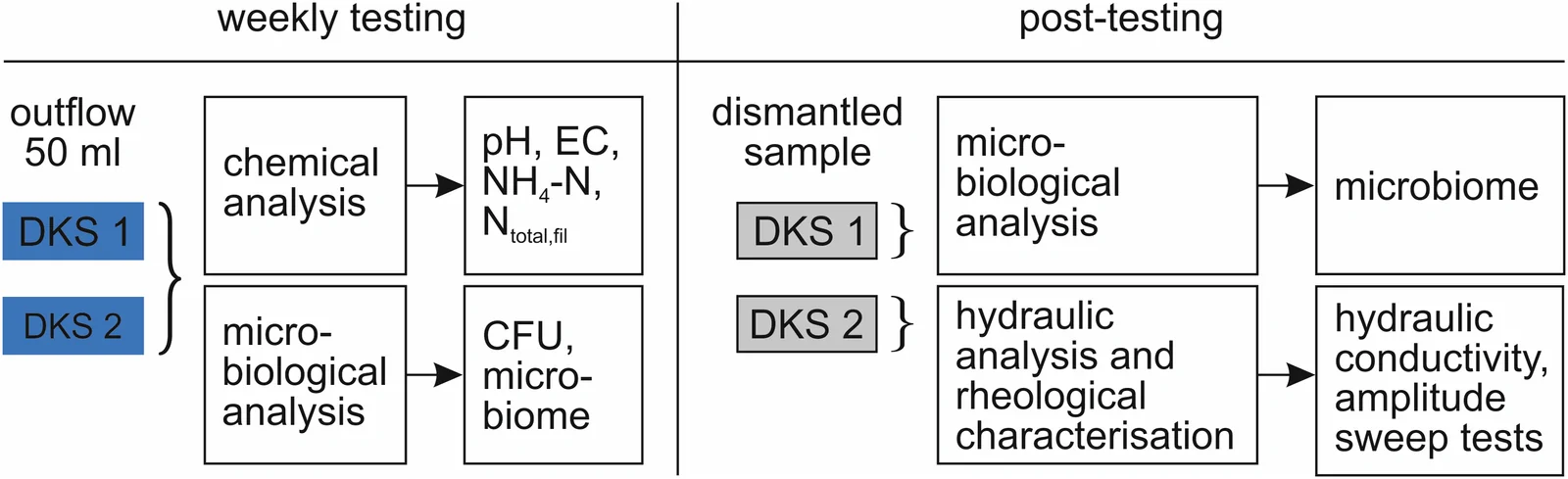
Overview of the material investigations conducted.

### Determination of the polymer content in the polymer-modified samples

The polymer content of the treated soils was not measured directly but estimated using two independent approaches: (i) loss-on-ignition (LOI) analysis of the sediment (paste) and supernatant (excess liquid) fractions, and (ii) rheological characterisation of the supernatant. The combined evaluation of both datasets allows an estimation of the initial polymer content in the polymer-modified MX80.

The LOI was determined following EN 17685−1. All measurements were conducted in triplicate to obtain reliable average values and to assess the variability of the method. The polymer content in the sediment and supernatant was calculated as ratio of polymer mass to dry soil mass using:


$$\begin{array}{c} \frac{m_{d,\;Polymer}}{m_{d,\;Sediment/Supernatant}}=\frac{{LOI}_{Sediment/Supernatant}-{LOI}_{Soil}}{{LOI}_{Polymer}-{LOI}_{Soil}}\;[-] \end{array}$$ (1)

where LOI_Soil_ and LOI_Polymer_ are the LOIs of the pure materials, and LOI_Sediment_ and LOI_Supernatant_ represent the LOIs of the polymer-soil-mixtures. The method assumes ideal additivity of mass loss between soil minerals and polymer.

To independently validate the LOI-derived polymer concentrations, the viscosity–shear rate behaviour of the supernatants was measured using an MCR 102e rheometer (Anton Paar) equipped with a double-gap cylinder geometry, suitable for viscosities between 1 and $1\cdot 10^{8}$ mPas. All measurements were performed at a controlled temperature of $25{\;}^{\circ }C$. The viscosity was recorded as a function of shear rate over the range $\gamma =10^{-1}\;\text{--}\;10^{-3}$ [1/s]. To establish calibration curves, flow curves of pure PAA^+^ and PAA^-^ solutions were measured at several known polymer concentrations. From these measurements, concentration–viscosity relationships were constructed. The specific viscosity was calculated according to:


$$\begin{array}{c} \eta_{sp}=\frac{\eta -\eta_{0}}{\eta_{0}}\;[mPa\cdot s] \end{array}$$ (2)

where η is the zero-shear viscosity, determined by simple plateau reading from the low-shear-rate region of the flow curve, and $\eta_{0}=1$ mPa·s denotes the viscosity of water.

### Chemical and microbiological analysis methods

#### Analytical methods.

The pH and EC were analysed according to DIN 38404−5 and DIN EN 27888, respectively. The chemical parameter NH_4_-N was determined using Hach-Lange cuvette tests LCK 303 and LCK 304. N_total,fil_ was quantified using Hach-Lange cuvette test LCK 238.

Microbiological analyses included the determination of the OD at a wavelength of 600 nm using a Nanodrop 2000c spectrophotometer (Peqlab). The CFU were determined by plating 50 μL of serial dilutions on BM agar plates (1.5% agar). The plates were incubated for 96 h at $28{\;}^{\circ }C$, after which the colonies were counted manually to calculate CFU mL^–1^. DNA extraction for subsequent 16S rRNA amplicon sequencing was performed using the PowerSoil Pro Kit (Qiagen). The sequencing of the 16S rRNA amplicons were conducted by an external analytical laboratory.

#### Outflow analysis.

Outflow samples were collected every two weeks and analysed for pH and EC according to DIN 38404−5 and DIN EN 27888, respectively. The chemical parameters NH_4_-N and N_total,fil_ were also determined. Microbiological monitoring was conducted at two-week intervals, including measurements of OD and CFU. In addition, 16S rRNA amplicon sequencing was performed on selected samples to characterise the microbial community composition.

#### Composite soil sample analysis.

Following completion of the outflow sampling, the soil samples from the DKS permeameter were subdivided into an 8 × 8 grid. Individual grid cells were assigned to eight composite samples using a rotating numerical scheme to obtain representative subsamples covering the entire cross-sectional area. 16S rRNA amplicon sequencing was conducted for the composite samples. For comparison and to assess the development of the bacterial class distribution during the experiment, the installed materials were also analysed by 16S rRNA amplicon sequencing.

### Hydraulic conductivity

Permeability tests were conducted on the soil samples taken from the DKS-permeameters in accordance with DIN EN ISO 17892−11 using a flexible-wall permeameter. Due to the low permeability of the material, the falling-head method was applied, with a constant tailwater level and an initial hydraulic gradient of i = 30. Hydraulic conductivity was determined using the standard evaluation equation. Samples were subjected to a vertical stress of 10 kPa, matching the stress applied during long-term testing in the DKS-permeameter. Permeability tests were performed after 32 weeks of DKS testing.

### Amplitude sweep tests

Amplitude sweep tests were conducted using an MCR 301 rheometer (Anton Paar) with a 40 mm plate–plate geometry. All measurements were conducted at a controlled temperature of $25{\;}^{\circ }C$. The gap size was set to 1 mm and trimmed after loading to ensure reproducible boundary conditions. Following sample placement, a resting time of 1 min was allowed to ensure structural relaxation before initiating the oscillatory measurements. The storage modulus $G^{\prime }$ and loss modulus $G^{\prime \prime }$ were recorded over a strain range of $\gamma =10^{-2}\%\;\text{--}\;10^{2}\%$. All tests were conducted at an angular frequency of ω=10rad/s. The linear viscoelastic (LVE) region was defined as the strain interval in which $G^{\prime }$ remained constant within a 5% deviation criterion, indicating that the internal microstructure was not disturbed under oscillatory loading. Consistent with the hydraulic permeability testing programme, all rheological measurements were performed after saturation in the DKS-permeameters and subsequent consolidation under a vertical effective stress of 10 kPa.

## Results

### Bacterial growth curves

The generation time of Pseudomonas, one of the polymer-degrading bacteria identified in MX80, was determined using PAA^+^ and PAA^-^ as the sole carbon and nitrogen sources. The generation time (g) corresponds to the time required for the bacterial population to double. To this end, growth curves were plotted using the parameters OD and CFU (Fig 5). The two parameters differ in that OD detects both living and dead organisms, whereas the CFU analysis considers only living organisms. It can be seen that, for both OD and CFU, the rise in the curves during the exponential growth phase is steeper for PAA^+^ than for PAA^-^. The generation times were calculated based on both the linear increase in OD and the exponential increase in CFU. The results show that Pseudomonas has a shorter generation time on PAA^+^ ($g_{CFU}$ = 6.3 h; $g_{OD}$ = 6.6 h) than on PAA^-^ ($g_{CFU}$ = 18 h; $g_{OD}$ = 11 h). The accelerated growth on PAA^+^ suggests that this polymer is degraded more readily, thereby making nutrients available more efficiently and supporting faster cell division. In contrast, the slower growth on PAA^-^ reflects a lower rate of polymer utilisation.

**Fig 5:**
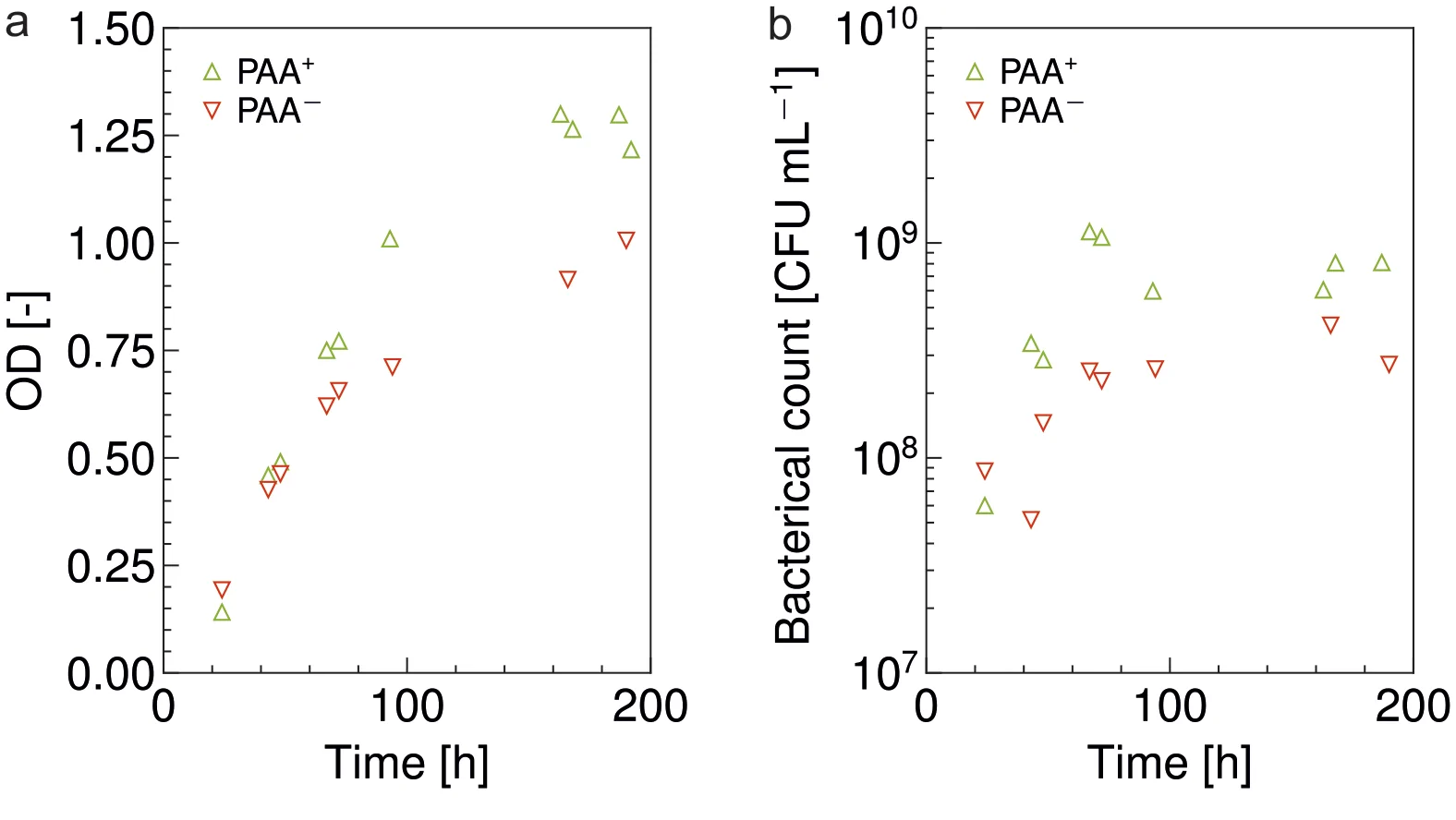
Growth curves of Pseudomonas. a: OD evolution. b: CFU evolution.

### Analysis of initial state of the polymer-modified samples

The LOI values of the reference materials (MX80, PAA^+^, and PAA^-^) and of the sediment and supernatant samples are summarised in Table 1. Using Eq (1), polymer contents of 5.2 wt% for PAA^+^ modified MX80 and 2.3 wt% for PAA^-^ modified MX80 were obtained for the sediment. However, [31] have shown that LOI-derived polymer contents can deviate from the actual values because thermal degradation of the polymer is influenced by interactions with the bentonite. Hydration of the bentonite-polymer composites further decreases the accuracy of LOI-based estimates. Therefore, the LOI-based estimates were compared with the known initial polymer-to-water mass ratios used during sample preparation. The ratios between LOI-derived total polymer content (i.e., the combined LOI contributions from sediment and supernatant) and the known initial polymer content lead to correction factors of 1.09 for MX80/PAA^+^ and 0.93 for MX80/PAA^-^. The corrected polymer contents are therefore 5.7 wt% and 2.1 wt%, respectively. Overall, the polymer content in the MX80/PAA^+^ system is approximately 2.7 times higher than in the MX80/PAA^-^ system. For the supernatant, polymer concentrations (expressed as g polymer per kg water) were determined analogously and corrected using the same factors. This yielded concentrations of 0.8 g/kg for MX80/PAA^+^ and 1.5 g/kg for MX80/PAA^-^ (Table 1).

**Table 1:** LOI and polymer contents in soil and water phases.

	LOI	Polymer/soil	Polymer/soil corrected	Polymer/water	Polymer/water corrected
	[%]	[wt %]	[wt %]	[g/kg]	[g/kg]
MX80	1.6	–	–	–	–
PAA^+^	98.7	–	–	–	–
PAA^-^	72.6	–	–	–	–
Sediment (MX80/PAA^+^)	6.6	5.2	5.7	–	–
Sediment (MX80/PAA^-^)	3.2	2.3	2.1	–	–
Supernatant (MX80/PAA^+^)	44.5	44.1	48.1	0.8	0.8
Supernatant (MX80/PAA^-^)	12.8	9.8	9.1	1.6	1.5

LOI denotes loss on ignition. Corrected values account for systematic deviations identified during post-processing.

This observation is consistent with the findings of [16], who reported that PAA^+^ interacting with MX80 exhibits significantly higher maximum adsorption capacities than MX80/PAA^-^ systems prepared using the solution intercalation method. [16] attributes this difference to the underlying adsorption mechanisms: cation bridging on multivalent exchangeable cations, which governs anionic polymer adsorption, occurs in much smaller quantities than cation exchange, the dominant mechanism for cationic polymer adsorption.

Fig 6 displays the viscosity–shear rate relationships of the supernatants together with those of pure polymer solutions at different concentrations. The resulting calibration curves (Fig 7) show a linear relationship between specific viscosity $\eta_{sp}$ and polymer concentration, with a correlation coefficient of R^2^ = 0.99 for both polymers. Using the specific viscosities determined from the supernatant flow curves and the calibration functions, rheology-based polymer concentrations of $c^{*}=1.2\cdot 10^{-2}$ g/kg (MX80/PAA^+^) and $c^{*}=8.4\cdot 10^{-1}$ g/kg (MX80/PAA^-^) were obtained. Comparison with the LOI-derived concentrations reveals substantial deviations: the rheological estimates correspond to 1.5% (MX80/PAA^+^) and 55.8% (MX80/PAA^-^) of the LOI-derived values. This discrepancy may reflect differences in the effective molar mass distribution of the polymers remaining in suspension. High-molecular-weight polymer fractions, which dominate the zero-shear viscosity response, may preferentially adsorb onto or intercalate into the bentonite microstructure. Based on this assumption, the supernatant becomes enriched in low-molecular-weight fractions, which contribute significantly less to viscosity. Thus, rheology may predominantly capture the effective chain-length distribution of the dissolved polymer, whereas LOI quantifies the total polymer mass, regardless of molecular size. It should be noted that the discrepancy between the rheologically derived estimates and the LOI-based values is less pronounced for MX80/PAA^-^ than for MX80/PAA^+^. This could indicate that, in the MX80/PAA^-^ system, comparatively high-molecular-weight polymer fractions remain in the supernatant, whereas the polymer fraction retained in the sediment likely consists, on average, of lower-molecular-weights compared to MX80/PAA^+^. Although no direct molar mass distribution characterisation was performed in this study, the proposed mechanism provides a plausible explanation for the observed differences between the rheological and LOI-based estimates.

**Fig 6:**
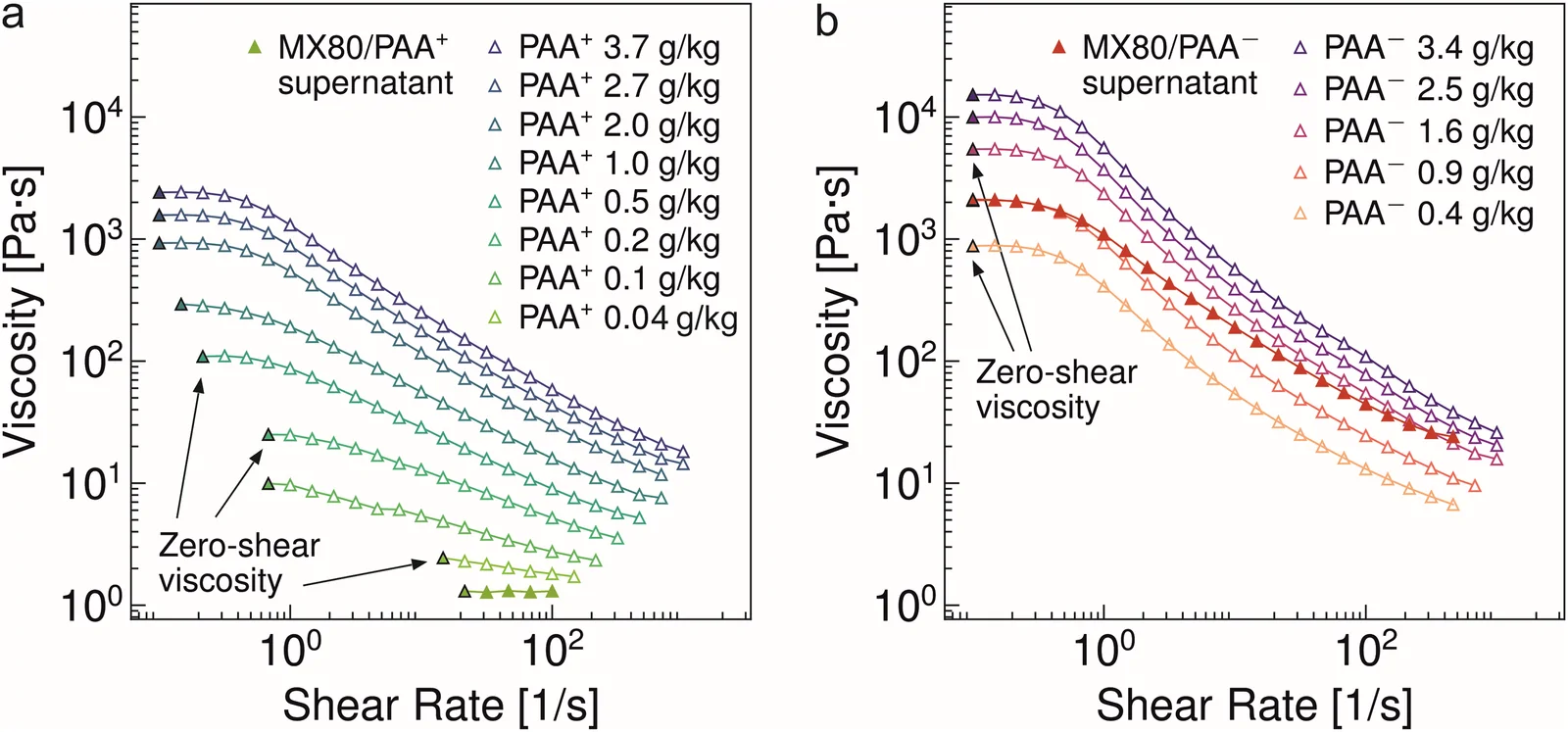
Viscosity–shear rate relationships of the supernatants and the corresponding pure polymer solutions at different concentrations. a: Cationic polymer PAA^+^. b: Anionic polymer PAA^-^.

**Fig 7:**
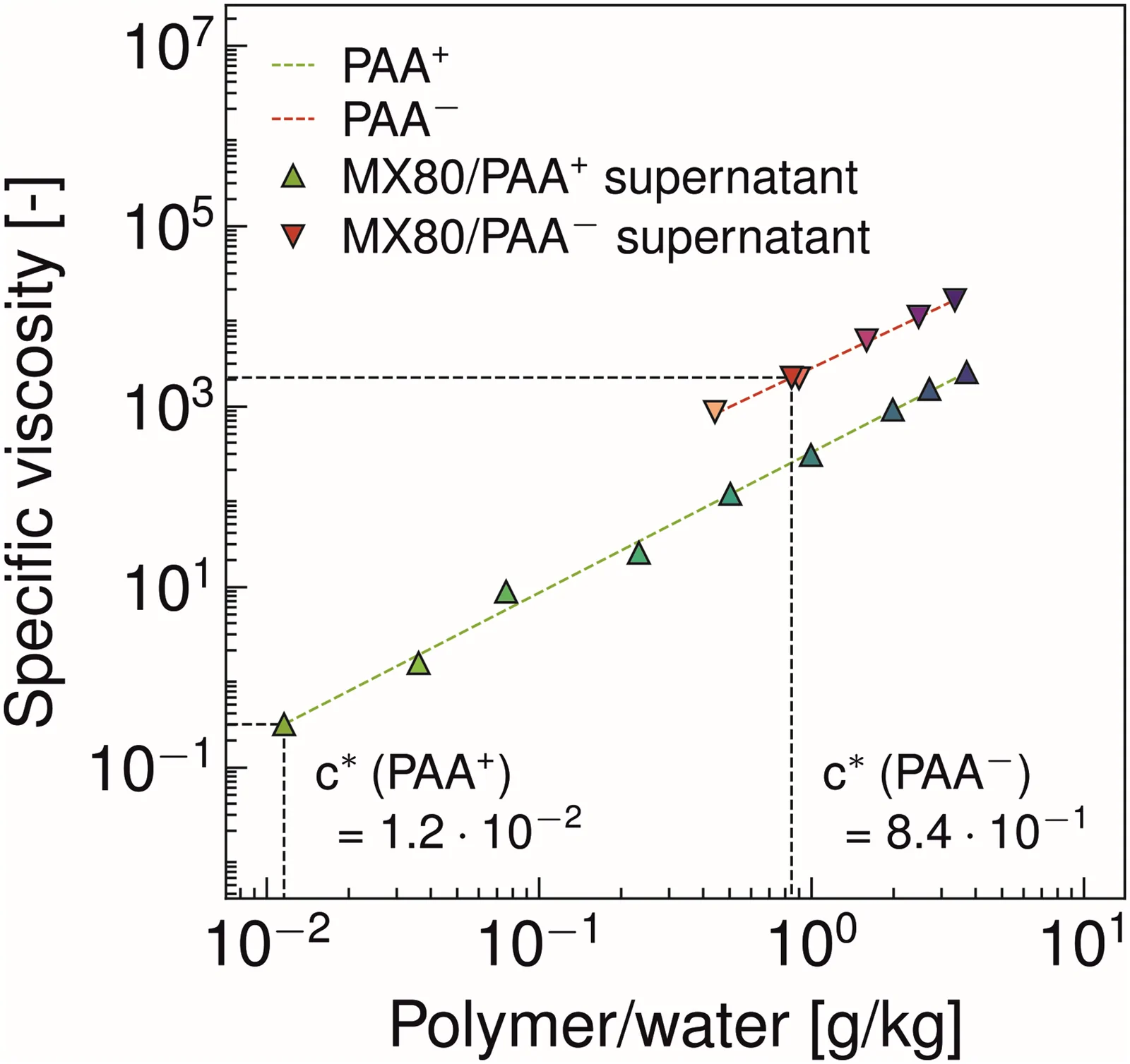
Specific viscosities derived from the viscosity–shear rate of the supernatants and pure polymer solutions as a function of polymer concentration.

### Chemical response of the outflows

Monitoring both pH and EC provides a comprehensive assessment of the degradation behaviour of polymer-modified soils. While pH primarily reflects the release of acidic degradation products, EC indicates the overall concentration of ions mobilised during polymer breakdown and from soil-polymer interactions. Assessing both parameters simultaneously therefore offers a more complete understanding of polymer biodegradation, and their influence on soil-water chemistry. Fig 8a shows the temporal evolution of pH in the outflows over 32 weeks of testing for all samples variants. The pH of the pure MX80 reference remained stable between 7.4 and 7.8 throughout the whole experiment. In contrast, both polymer-modified systems exhibited a clear shift toward acidic conditions. In the MX80/PAA^+^ samples, pH decreased to 5.5 within the first eight weeks, remained constant for six weeks, and then slightly increased to pH 6.5 for the remainder of the test. MX80/PAA^-^ showed a slower decline, reaching pH 6.3 after twelve weeks and remaining stable thereafter. These results suggest that both polymers may release acidic degradation products in the initial phase, with PAA^+^ degrading more quickly. Additionally, microorganisms utilise the polymer as a carbon source and release CO_2_ as a metabolic by-product. When this CO_2_ dissolves in pore water, it forms carbonic acid (H_2_CO_3_), which may also contribute to the initial decrease in pH. Although a slight rebound in pH in PAA^+^ is observed during the stabilisation phase, the values remain clearly below those of the reference sample, indicating that degradation is still ongoing. This suggests that the dominant degradation occurs initially, followed by a slower but persistent degradation process.

**Fig 8:**
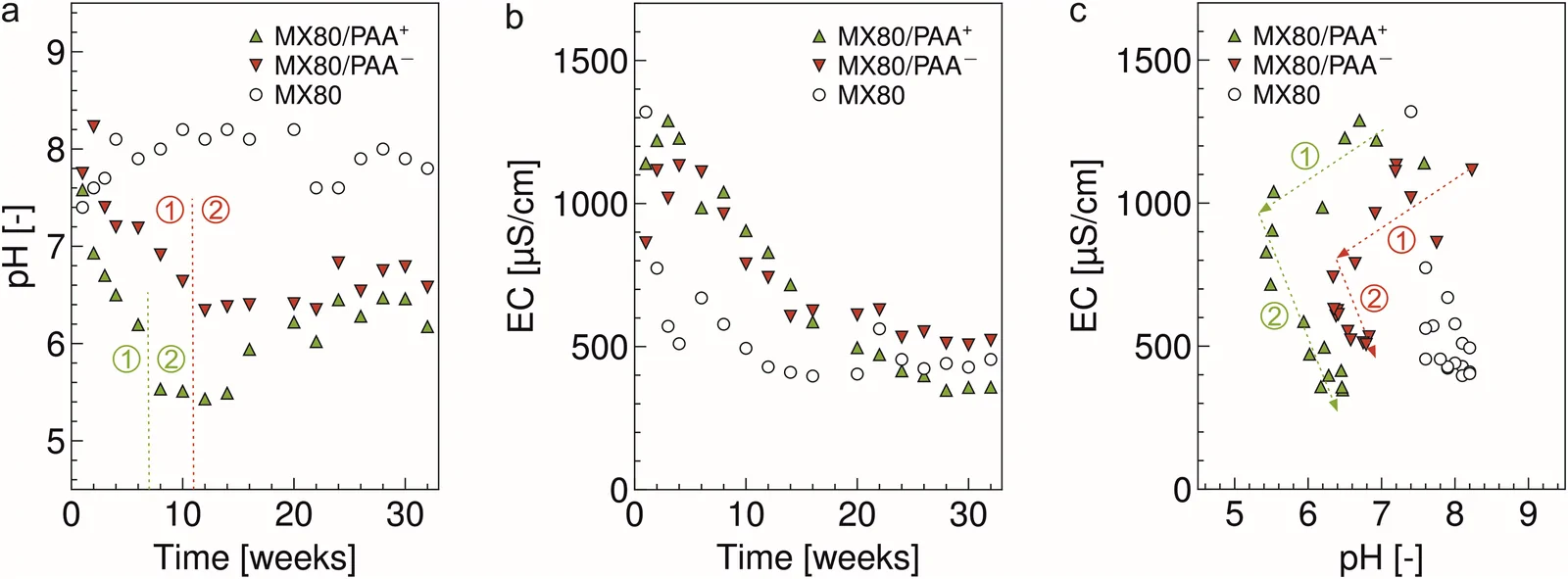
Temporal evolution of pH and EC in the outflows over 32 weeks. a: pH evolution. b: EC evolution. c: Relationship between EC and pH.

The EC of the outflows shown in Fig 8b provides additional information on ion mobility that is not captured by pH alone. Initially, MX80 exhibited an EC of 1320 μS/cm, while MX80/PAA^+^ and MX80/PAA^-^ showed lower values of 1140 μS/cm and 863 μS/cm, respectively, suggesting reduced ion release due to polymer addition. During the first four weeks, the EC of MX80 decreased to 510 μS/cm and stabilised at 400 – 500 μS/cm. In contrast, polymer-modified soils initially showed an EC increase to 1590 μS/cm (MX80/PAA^+^) and 1130 μS/cm (MX80/PAA^-^), followed by a decline to 415 μS/cm and 533 μS/cm, respectively, by week 24. This transient EC rise likely reflects the release of ions associated with polymer degradation. Together with the pH evolution, these observations indicate that polymer degradation is most active during the initial weeks, after which the system reaches a steady-state with stable pH and ion concentrations.

Fig 8c shows a non-linear EC–pH relationship for the polymer-modified systems, displaying two distinct behavioural regimes. In the initial stage ①, decreasing EC with decreasing pH is observed, indicating decreasing ion release and decreasing solution reactivity. This trend is followed by a second stage ②, in which EC decreases despite renewed increase in pH, suggesting a shift in the dominant controlling processes. The presence of these two regimes points to a potentially multi-stage system response rather than a single continuous process. Such behaviour may reflect successive biochemical mechanisms, possibly associated with different microbial populations or metabolic pathways becoming dominant over time. In contrast, the unmodified MX80 system shows a comparatively narrow and stable EC–pH domain without evidence of stage-dependent behaviour. The transition between these regimes occurs between weeks 6 and 8 for PAA^+^ and between weeks 10 and 12 for PAA^-^, as identified by correlating the EC–pH data (Fig 8c) with the temporal evolution of EC and pH (Fig 8a,b). This transition point is indicated in Fig 8a and corresponds to the time at which the previously observed decrease in pH for both PAA^+^ and PAA^-^ levels off.

To complement the chemical characterisation based on pH and EC, the cumulative NH_4_-N and N_total,fil_ loads were determined. While pH and EC reflect the general release of acidic and ionic species, measuring NH_4_-N and N_total,fil_ captures the nitrogen-containing solutes in the leachate, including ammonium associated with amide bond hydrolysis and dissolved short-chain polymer fragments. Accordingly, these parameters provide an integrated measure of nitrogen mobilisation within the system. As shown in Fig 9a and b, the cumulative NH_4_-N and N_total,fil_ leached from polymer-modified soils were substantially higher than from the unmodified MX80, which exhibited only minimal leaching. This confirms that the observed nitrogen release is predominantly derived from the polymer.

**Fig 9:**
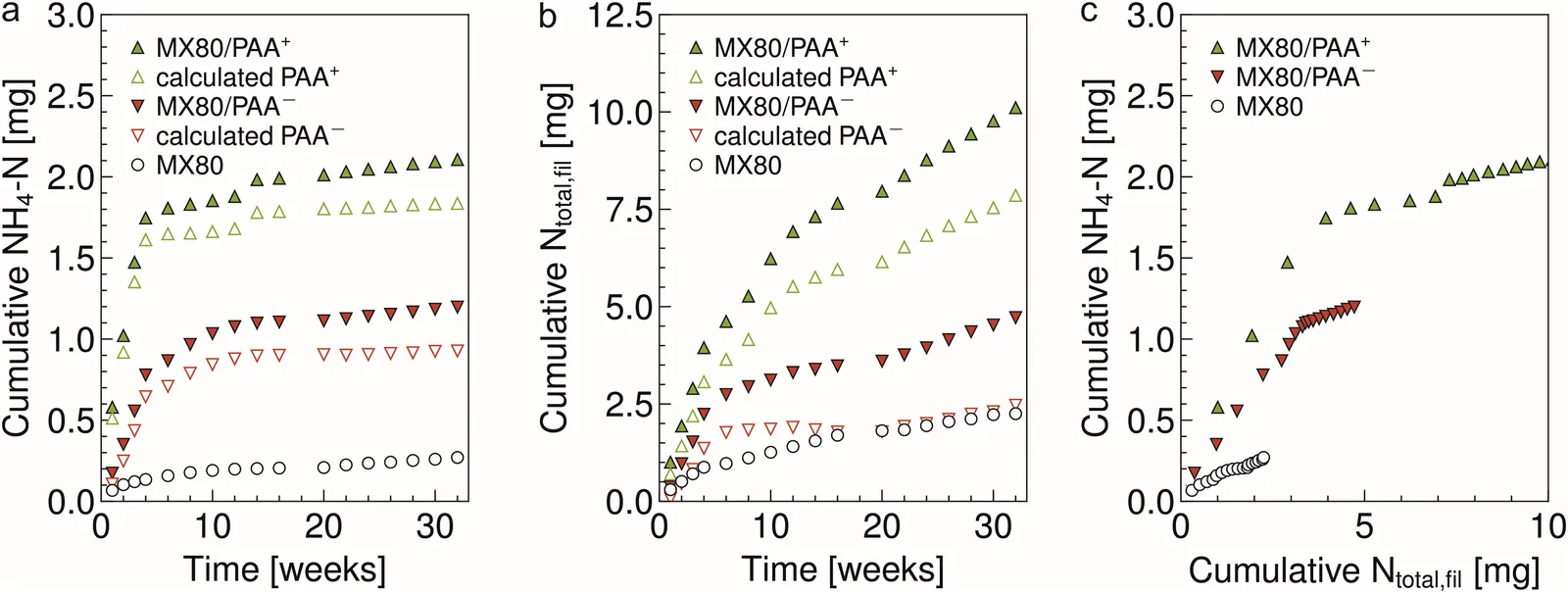
Cumulative NH_4_-N and N_total,fil_ release in the outflows over 32 weeks. Calculated curves represent polymer-only contributions after subtracting baseline MX80 leaching. a: NH_4_-N release. b: N_total,fil_ release. c: Relationship between NH_4_-N and N_total,fil_.

For unmodified MX80, the cumulative NH_4_-N load across 17 sampling events was 0.27 mg, with 0.07 mg occurring in the first week and the rate steadily declining thereafter (Fig 9a). In contrast, the outflow of the PAA^+^ modified MX80 showed the highest NH_4_-N release, with approximately 0.5 mg in each of the first three samples and a total of 2.1 mg. The MX80/PAA^-^ system released ~0.2 mg in the first four samples and ~0.1 mg in the subsequent two, in total 1.2 mg. These values clearly exceed those of the unmodified soil and therefore originate from the polymer. However, it is not possible to conclusively determine whether the measured NH_4_-N originates from polymer degradation or from primary amine groups associated with the monomer, since colorimetric cuvette tests (Hach Lange) detect both free ammonium and primary amines. The latter are present in the monomer acrylamide (prop-2-enamide) at the same ratio in both polymers. However, the ionic monomers themselves, which carry either tertiary amine (PAA^+^) or carboxylate (PAA^-^) groups, are unlikely to cause direct analytical interference. This suggests that the results for MX80/PAA^+^ and MX80/PAA^-^ are comparable despite potential methodological limitations. N_total,fil_ followed a similar trend (Fig 9b), indicating that additional nitrogen sources, beyond NH_4_-N, were leached. Total nitrogen loads were 10 mg for MX80/PAA^+^, 4.7 mg for MX80/PAA^-^, and 2.3 mg for unmodified MX80.

In Fig 9c, the cumulative N_total,fil_ is plotted against the cumulative NH_4_-N. While the unmodified MX80 sample exhibits a linear relationship across all sampling events, two distinct regimes can be identified for the polymer-modified samples. For PAA^+^, a pronounced increase in NH_4_-N is observed during the first four sampling events, followed by a markedly reduced increase in NH_4_-N relative to N_total,fil_. In contrast, this transition point occurs only after the seventh sampling event for PAA^-^. It should be noted that the reduced increase in NH_4_-N after the transition point is comparable for both polymer-modified samples and closely matches the slope observed for the unmodified MX80. This indicates that during the initial regime, NH_4_-N release is primarily associated with polymer biodegradation. Beyond the transition point, however, NH_4_-N may likely originate from leaching of native soil nitrogen or microbial biomass and is not necessarily linked to ongoing polymer degradation. Notably, the identified transition points are consistent with those derived from the EC-pH relationship.

### Nitrogen balance

To close the nitrogen balance, the cumulative NH_4_-N and N_total,fil_ loads were corrected by subtracting the NH_4_-N and N_total,fil_ washed out of unmodified MX80. This yields adjusted NH_4_-N releases of 1.8 mg (calculated pure PAA^+^) and 0.9 mg (calculated pure PAA^-^), and adjusted N_total,fil_ loads of 7.9 mg (calculated pure PAA^+^) and 2.5 mg (calculated pure PAA^-^). The difference of the corrected values between N_total,fil_ and NH_4_-N therefore represents the fraction of nitrogen that is not present as NH_4_-N and thus attributable to other nitrogen compounds. For PAA^+^, this difference is 6.0 mg (7.9 mg−1.8 mg), while for PAA^-^ it amounts to 1.5 mg (2.5 mg−0.9 mg). For PAA^+^, the contribution of nitrogen from the positively charged monomer must be considered. Of the N_total,fil_ released (6.0 mg), 1.2 mg can be attributed to the cationic monomer. This leaves a surplus of 4.8 mg (6.0 mg–1.2 mg) that is not directly linked to the polymer itself. The remaining nitrogen may exist in both organic and inorganic forms. However, a significant portion likely originates from leached microbial biomass. It should be noted that this allocation is based on indirect evidence derived from mass balance and has not been independently verified by specific biomass quantification methods. Nevertheless, the microbial contribution to this nitrogen release is supported by the independent growth experiments (Fig 5), which showed that polymer-degrading microorganisms exposed to PAA^+^ exhibited generation times two to three times higher than those exposed to PAA^-^. This indicates that the additional nitrogen released in the presence of PAA^+^ is closely associated with microbial activity, as the ratio of nitrogen derived from PAA^+^ to PAA^-^ compounds is similarly elevated by approximately a factor of three.

To complement chemical nitrogen analyses (NH_4_-N and N_total,fil_) and to assess the contribution of microbial biomass to nitrogen release, microbial washout was quantified as CFU concentrations (CFU ${mL}^{-1}$). In unmodified MX80, CFU concentrations remained around 10^6^ CFU ${mL}^{-1}$ over the entire test period (Fig 10). Polymer-modified soils exhibited higher concentrations during the first two weeks ($3-5\cdot 10^{6}$ CFU ${mL}^{-1}$), followed by a pronounced peak in weeks 3–4 (up to $2\cdot 10^{8}$ CFU ${mL}^{-1}$ for MX80/PAA^+^ and $5\cdot 10^{8}$ CFU ${mL}^{-1}$ for MX80/PAA^-^), and a decline to MX80 levels from week 6 onwards.

**Fig 10:**
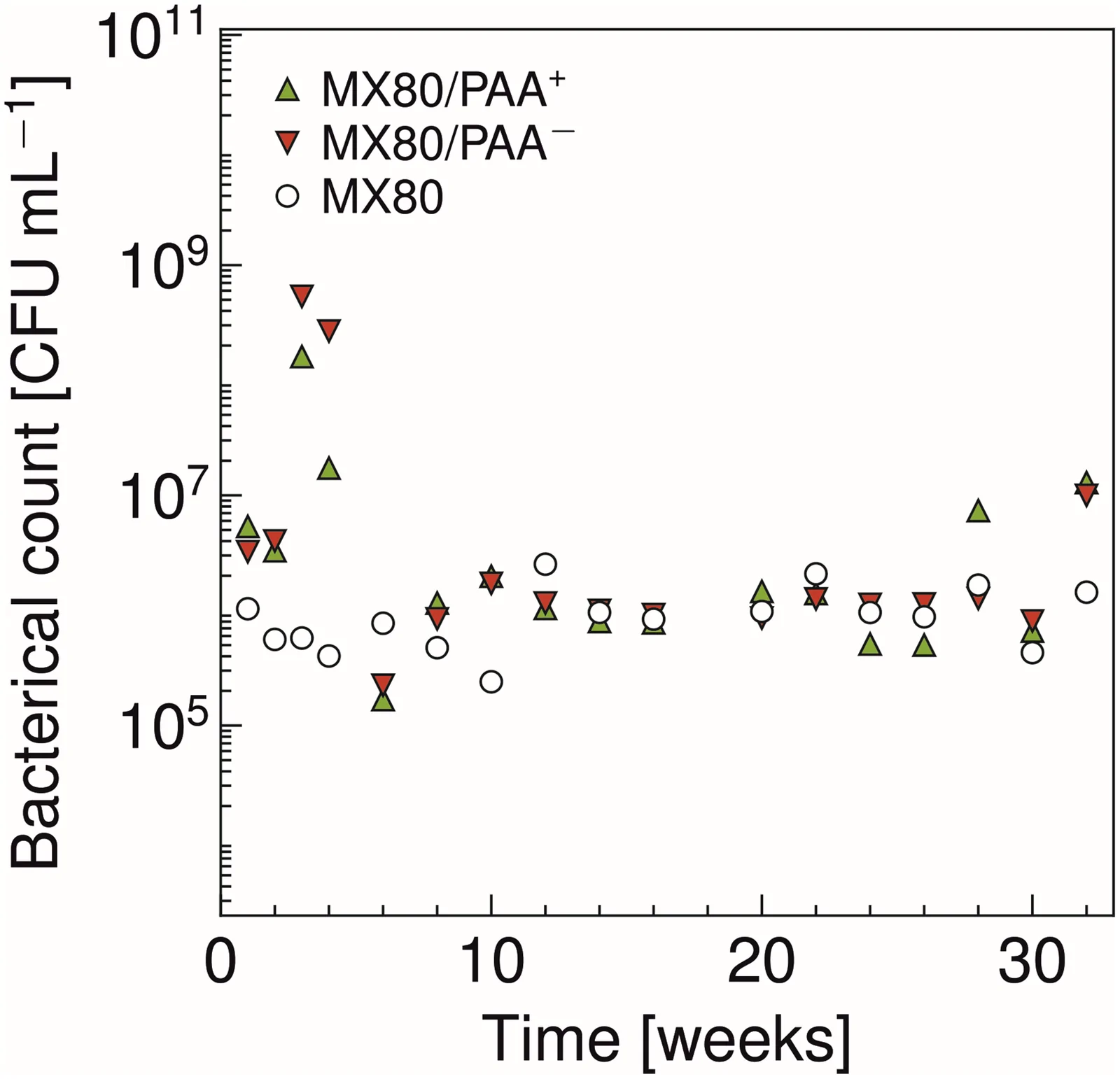
Cumulative CFU in the outflows over 32 weeks.

### Biocenosis analysis of polymer-modified samples and outflows

While CFU measurements quantify the abundance of viable microorganisms, they provide limited information on the taxonomic composition or functional potential of the community. Similarly, chemical nitrogen analyses capture only the overall nitrogen flux but cannot identify the taxa involved in degradation processes. To overcome these limitations, 16S amplicon analyses were performed, enabling detailed characterisation of the biocoenosis in the installed and dismantled materials as well as selected outflows.

The installed polymer-modified samples showed a heterogeneous bacterial distribution. Comparison of installed and dismantled samples revealed substantial shifts in community composition (Fig 11). Proteobacteria (Alpha-, Beta-, and Gammaproteobacteria), including polymer-degrading Pseudomonas, accounted for 74 – 92% of the installed material but decreased to 24% and 11% in the dismantled samples. As obligate aerobes, most Proteobacteria could not persist under the experimental conditions. In contrast, obligate anaerobic Clostridia increased from 0 – 5% to 40% (MX80/PAA^+^) and 73% (MX80/PAA^-^), strongly impacting the ecosystem. Bacilli, facultative anaerobes, increased in MX80/PAA^+^ but decreased in MX80/PAA^-^, indicating better metabolic adaptation to PAA^+^.

**Fig 11:**
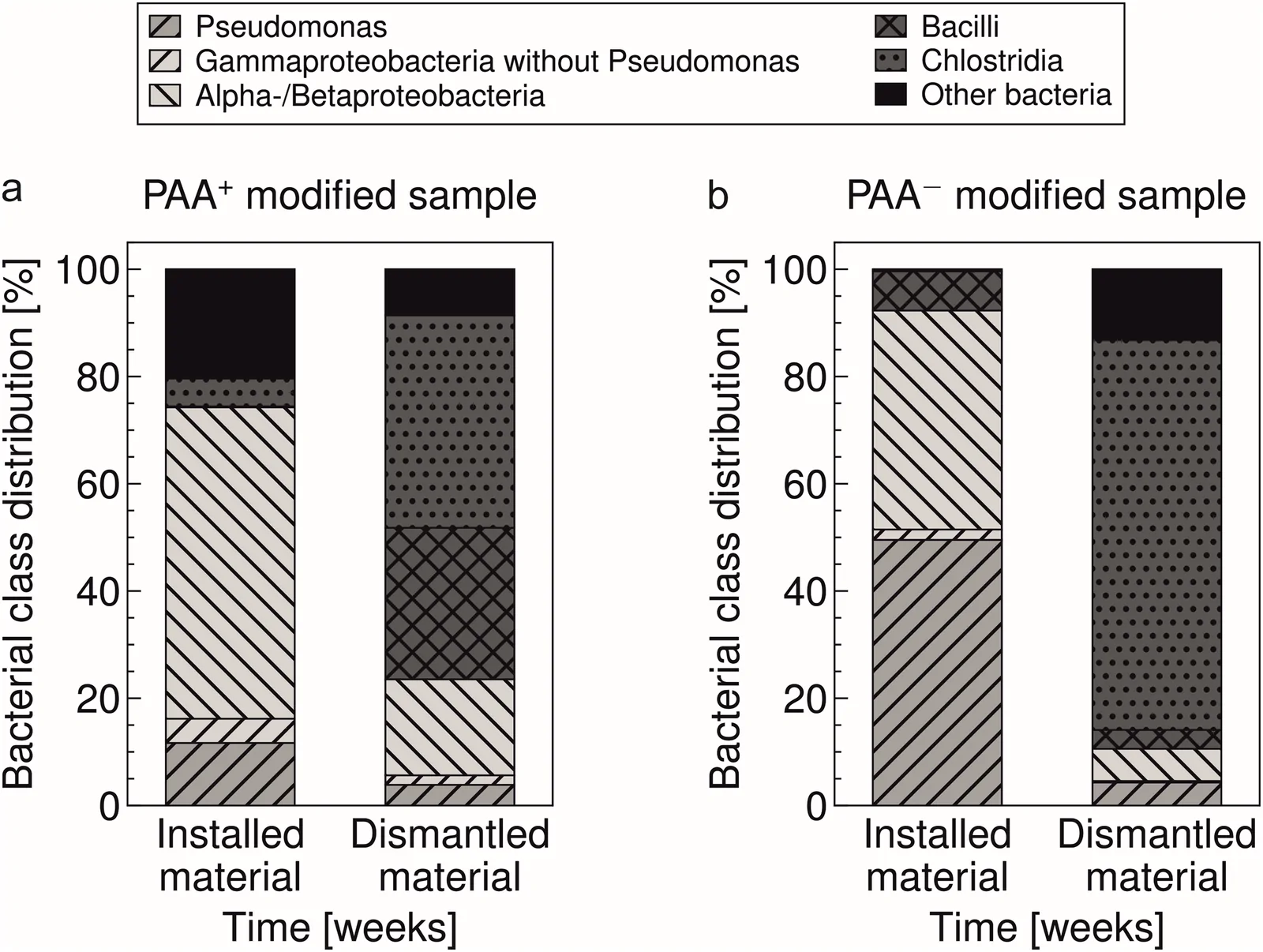
Comparison of the distribution of bacterial classes between installed and dismantled material for the polymer-modified samples. a: Cationic polymer PAA^+^. b: Anionic polymer PAA^-^.

Consistent with the shifts observed in the installed and dismantled materials, 16S rRNA gene amplicon analysis of the outflows reveals the temporal dynamics of Gammaproteobacteria and Pseudomonas (Fig 12). After one week, Gammaproteobacteria accounted for 9.8% (PAA^+^) and 4.2% (PAA^-^) of the total, with Pseudomonas accounting for 7.7% and 2.6% respectively. By week eight, both taxa had increased substantially to reach 56% and 17% for PAA^+^ and 42% and 14% for PAA^-^, respectively. At weeks 20 and 30, their relative abundances declined to below 2% and 1%, indicating that microbial proliferation was largely restricted to the initial phase of testing (<20 weeks).

**Fig 12:**
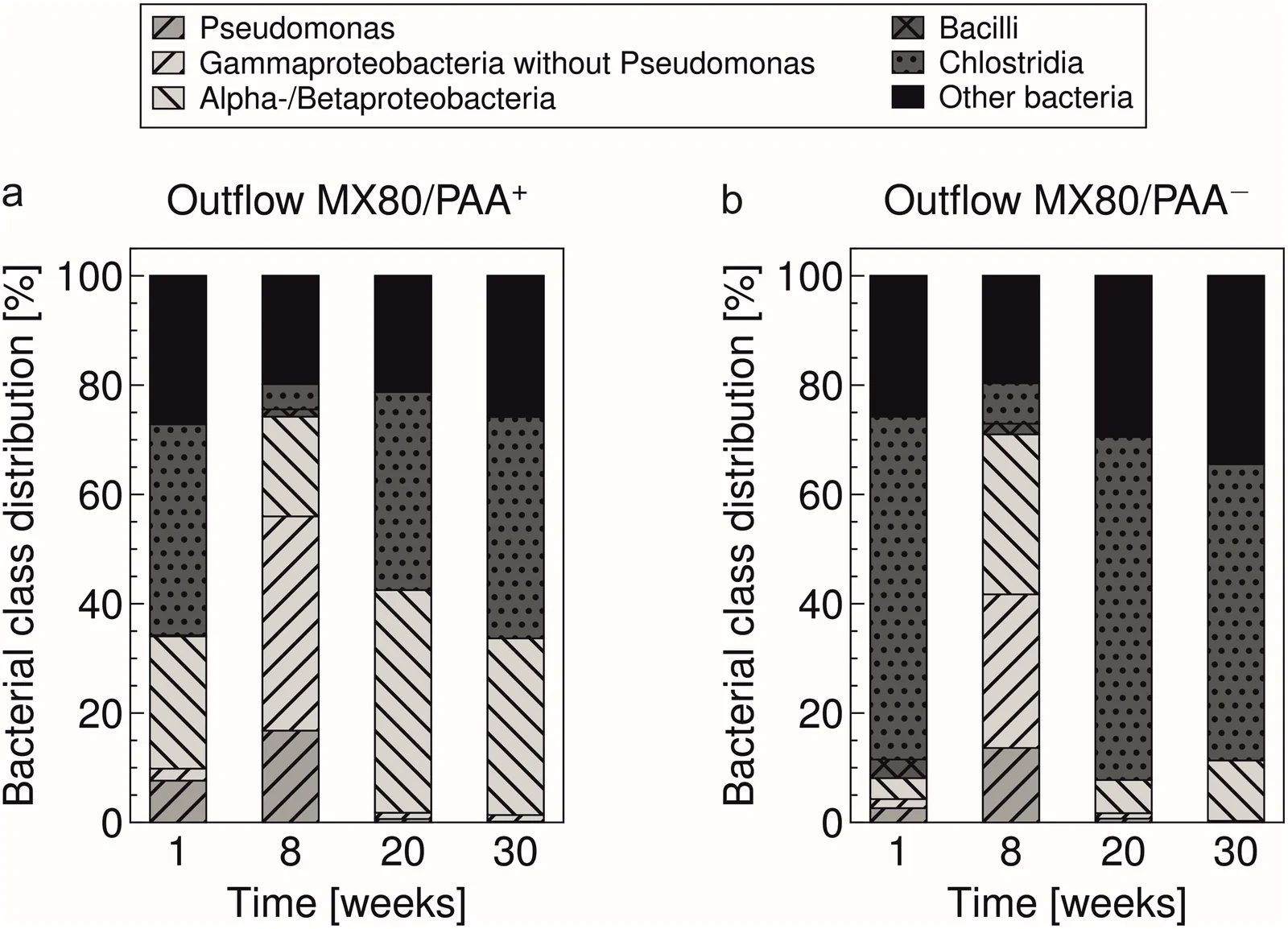
Development of the temporal distribution of bacterial classes for the outflow of the polymer-modified samples. a: Cationic polymer PAA^+^. b: Anionic polymer PAA^-^.

This temporal pattern corresponds to the identification of the two distinct regimes, as inferred from the EC–pH relationship (Fig 8c). Integrating the EC and pH data with the temporal distribution of bacterial classes (Fig 12) indicates that the initial increase in aerobic Proteobacteria may have induced a decrease in EC and pH (stage ①). In contrast, the subsequent increase in anaerobic Chlostridia bacteria, accompanied by a decline in aerobic taxa, may have led to a continued decrease in EC but a renewed increase in pH (stage ②). This interpretation is further supported by the sharp increase in cumulative ammonium release (Fig 9) and the peaks in CFU counts (Fig 10) within the first six weeks of testing. This suggests that there was initially sufficient dissolved or bound oxygen available to enable aerobic and facultative microorganisms to degrade the polymers. Presumably as a result of increasingly oxygen-limited conditions, nutrient turnover subsequently declined, culminating in a distinct regime transition between weeks 11 and 12 of testing. Continuous monitoring of dissolved oxygen and redox potential over the 32-week experimental period would allow verification of the processes underlying the observed microbial community shift.

### Hydraulic analysis of the dismantled samples

Fig 13a shows the temporal evaluation of the measured hydraulic conductivities of unmodified and PAA modified MX80 after dismantling. Based on the measured average values, the hydraulic conductivities followed the ascending order ($k_{MX80/PAA^{-}}<k_{MX80}<k_{MX80/PAA^{+}}$), with mean values of 1.9$\cdot 10^{-10}$ m/s, 3.6$\cdot 10^{-10}$ m/s, and 6.1$\cdot 10^{-10}$ m/s, respectively. Fig 13b presents the average hydraulic conductivities derived from these measurements as a function of void ratio. For comparison, reference data reported by [29], representing the initial hydraulic conductivity of the installed materials MX80 and MX80/PAA^+^, are included. The unmodified initial MX80 exhibits an increase in hydraulic conductivity with increasing void ratio. At a given void ratio, the dismantled MX80 shows a slight increase in hydraulic conductivity compared to the initial material. The initial PAA^+^ modification was found to increase the hydraulic conductivity of the initial MX80. This is likely because the PAA^+^ modification promotes a more open or aggregated clay fabric [29], thereby increasing the effective pore space available for flow. Furthermore, the dismantled MX80/PAA^+^ exhibits a slight increase in hydraulic conductivity compared to the initial MX80/PAA^+^. Importantly, the increase in hydraulic conductivity between the initial and dismantled states is of comparable magnitude for both sample types (MX80 and MX80/PAA^+^). This suggests that fabric reorganisation or the formation of preferential flow paths occurs during 32 weeks of testing. For MX80/PAA^-^, it is assumed in [16] that polymer modification does not significantly affect the pore structure and hydraulic conductivity. Therefore, the hydraulic conductivity of the initial MX80 was considered as a reference for evaluating the hydraulic behaviour of the dismantled MX80/PAA^-^. After 32 weeks of testing, the PAA^-^-modified specimen showed an approximately one-order-of-magnitude increase in hydraulic conductivity, likely due to polymer degradation and altered flow pathways.

**Fig 13:**
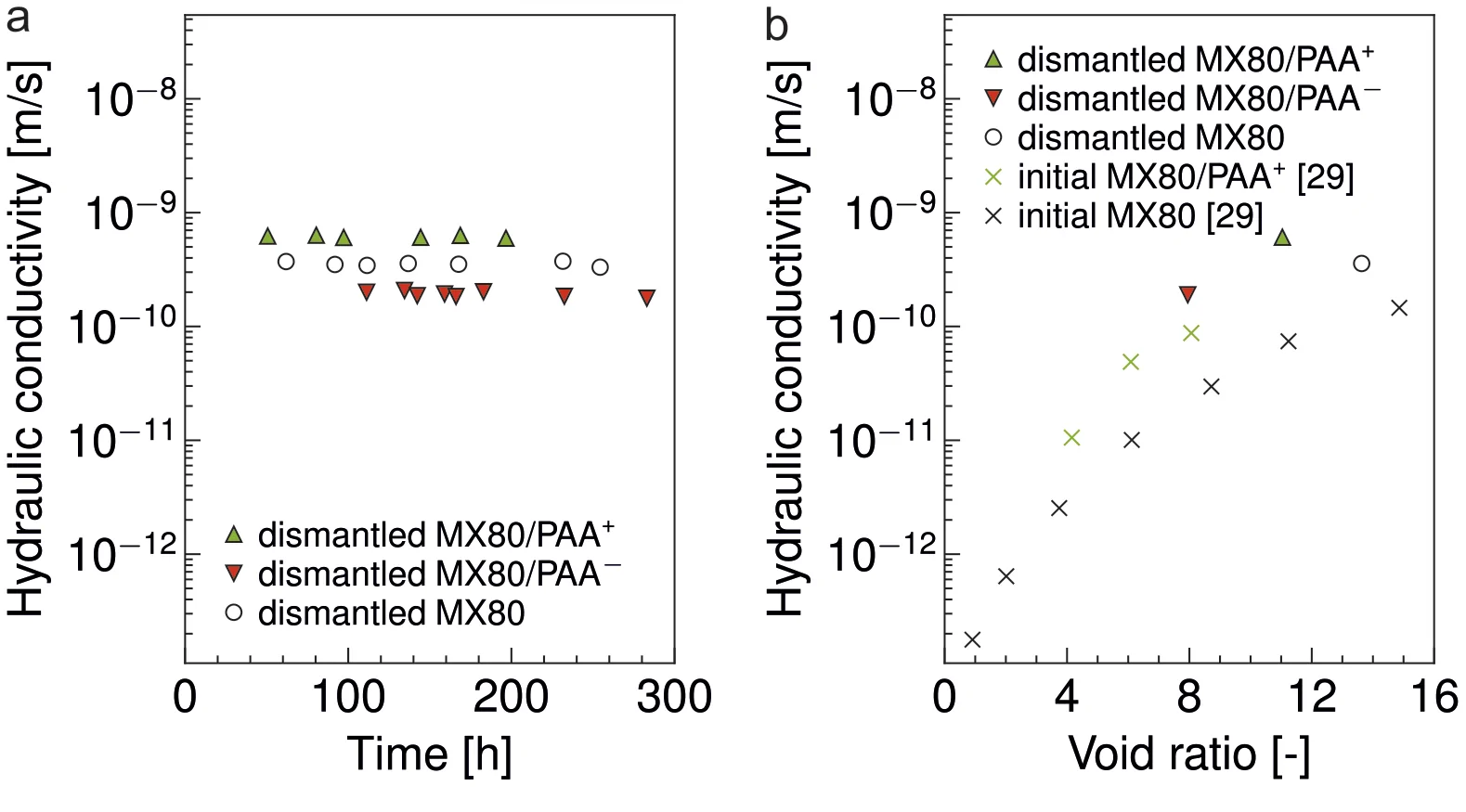
Results of hydraulic conductivity measurements. a: Temporal evolution of the hydraulic conductivity of dismantled samples. b: Hydraulic conductivity as a function of void ratio, comparing the initial state with dismantled material after 32 weeks of testing.

### Amplitude sweep tests of the dismantled samples

The amplitude sweep test conducted after 32 weeks provides key insights into the viscoelastic evolution and potential biodegradation of polymer-modified MX80 bentonite. Fig 14a and b show the storage modulus, $G^{\prime }$, and the loss modulus, $G^{\prime \prime }$, respectively, as function of shear strain for unmodified MX80 and polymer-modified samples. The storage modulus $G^{\prime }$ represents the elastic, energy-storing component of the material response, whereas the loss modulus $G^{\prime \prime }$ characterises the viscous, energy-dissipating component.

**Fig 14:**
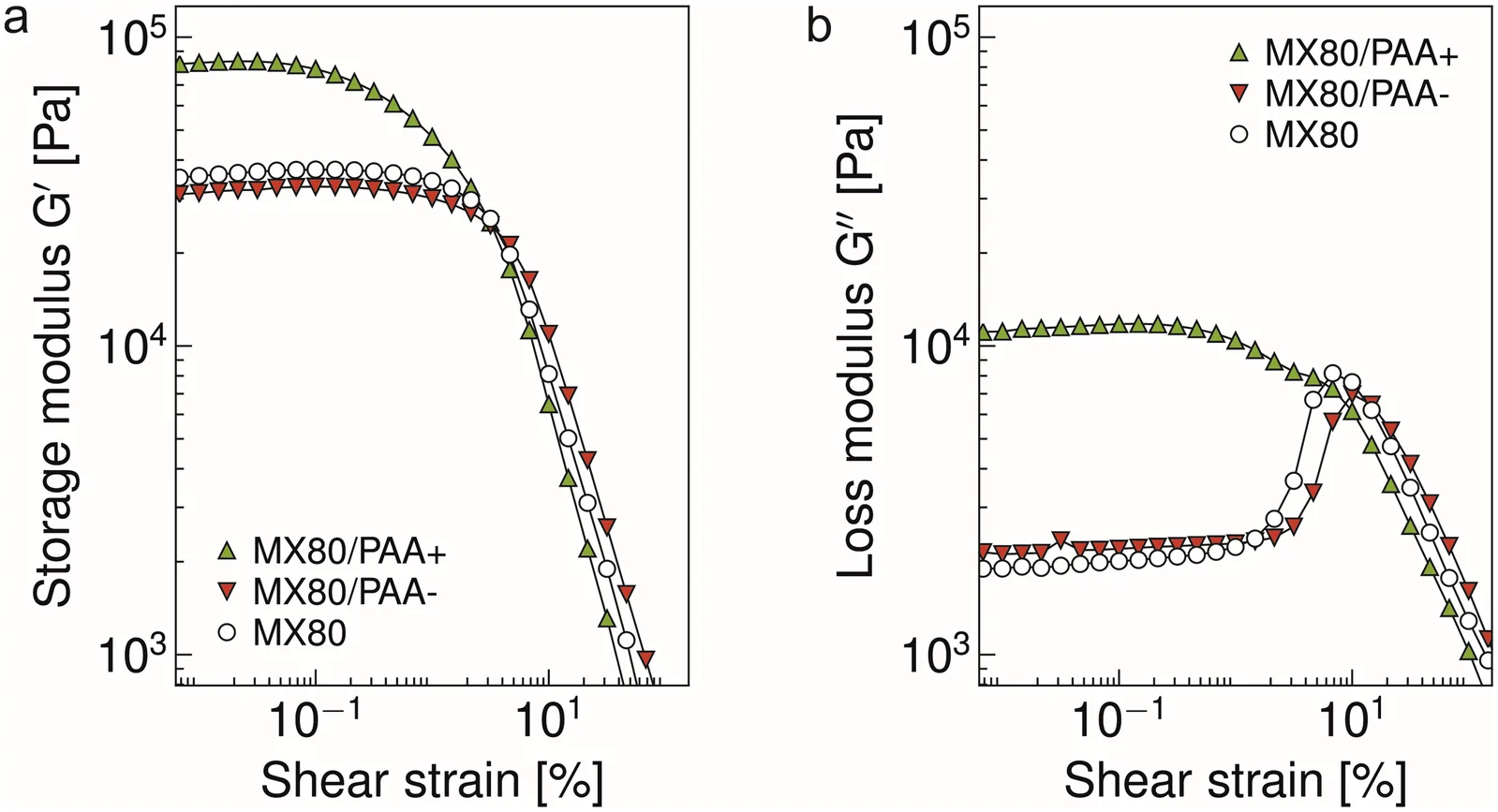
Amplitude sweep test results of unmodified MX80 and polymer-modified samples after 32 weeks of testing. a: Storage modulus. b: Loss modulus.

From the amplitude sweep curves, the linear viscoelastic (LVE) region can be identified as the strain range over which both $G^{\prime }$ and $G^{\prime \prime }$ remain constant, indicating a reversible and strain-independent response. The parameters $G_{0}^{\prime }$ and $G_{0}^{\prime \prime }$ denote the storage and loss moduli within this LVE regime, respectively. In addition, the ratio of loss to storage modulus defines the loss factor, $\delta =G^{\prime \prime }/G^{\prime }$, which quantifies the relative contributions of viscous and elastic behaviour. A loss factor close to unity indicates pronounced viscoelasticity, while δ=0 corresponds to ideal elastic behaviour and δ→∞ to ideal viscous flow.

Previous studies by [32] showed that intact, flocculated, negatively charged polymer-clay composites typically exhibit an enhanced small-strain stiffness ($G_{0}^{\prime }$), a reduced LVE region, and a more gradual yielding behaviour compared to unmodified clays. Such characteristics indicate increased resistance to abrupt structural rearrangement. This behaviour is clearly observed for the MX80/PAA^+^ samples, which exhibit higher storage and loss moduli at low shear strains than the unmodified MX80 (Fig 14). These elevated moduli can be attributed to the formation of irregular polymer-clay aggregates [16], resulting in a denser and mechanically more stable microstructure under the applied normal stress of 10 kPa. In contrast, this stabilising effect is not observed for the MX80/PAA^-^ samples after long-term testing. Their rheological response closely resembles that of unmodified MX80, indicating a loss of polymer-induced structural reinforcement.

An evaluation of the loss factor calculated from $G_{0}^{\prime }$ and $G_{0}^{\prime \prime }$ shows that all samples exhibit predominantly viscous behaviour, with δ>1. However, the unmodified MX80 displays the highest loss factor (18.6), followed by MX80/PAA^-^ (14.9) and MX80/PAA^+^ (6.7). This trend indicates that polymer modification reduces the relative viscous contribution of the bentonite system, particularly for the intact PAA^+^ modification.

Further insight is obtained from the evolution of the loss modulus with increasing shear strain (Fig 14b). For unmodified MX80, a pronounced peak in $G^{\prime \prime }$ is observed, reflecting rearrangement of the edge-to-face particle network under shear. This response is characteristic of smectitic clays with strong interparticle interactions [33]. In contrast, the MX80/PAA^+^ sample suppresses this characteristic $G^{\prime \prime }$ peak even after 32 weeks of testing. Conversely, the MX80/PAA^-^ sample exhibits a reappearance of the $G^{\prime \prime }$ peak, closely matching the response of unmodified MX80. This convergence is consistent with degradation of the polymer PAA^-^ and a progressive reduction in polymer-induced effects on the clay.

## Discussion

### Conceptional degradation model

The combined chemical, microbiological, hydraulic, and rheological data are consistent with a conceptual model describing the microscale fabric degradation of polymer-modified MX80 bentonite (Fig 15). This model is based on observed trends across the different datasets and explicitly considers the initial polymer–clay interaction mechanisms (Fig 1). Although both systems were subjected to identical boundary conditions, the results suggest that cationic (MX80/PAA^+^) and anionic (MX80/PAA^-^) systems follow distinct degradation pathways.

**Fig 15:**
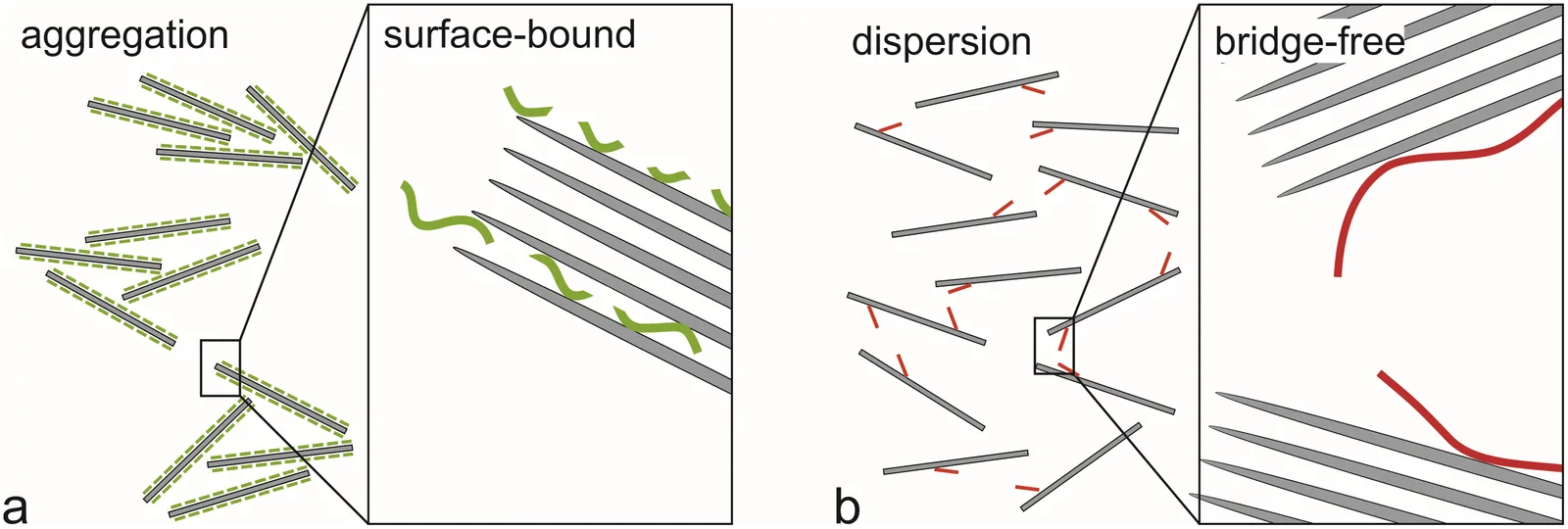
Degraded state of the microscale fabric of polymer modified bentonite. a: Clay-cationic polymer-composite. b: Clay-anionic polymer-composite.

Chemical and microbiological indicators reveal more pronounced degradation of PAA^+^ than PAA^-^, evidenced by a stronger pH decrease, higher EC peaks, and approximately twice the cumulative nitrogen release after 32 weeks (Figs 8, 9). Nevertheless, despite the enhanced degradation, the experimentally determined hydraulic conductivity of MX80/PAA^+^ shows only a slight increase after 32 weeks of testing (Fig 13). Furthermore, the viscoelastic response of the dismantled MX80/PAA^+^ is dominated by the PAA^+^ polymer network in the bentonite (Fig 14). This apparent contradiction may be explained by the initial fabric: PAA^+^ was present at higher effective concentrations (Table 1) and was firmly surface-bound (Fig 1). Furthermore, experimental polymer quantification by rheological measurements (Fig 7) and LOI analysis (Table 1) supports the model, indicating a comparably high-molecular-weight polymer fraction in MX80/PAA^+^. This fraction likely provides numerous adsorption points per polymer chain. Based on this, the model proposes that polymer-clay aggregates similar to the initial fabric are largely preserved, and that the aggregate-based structure may remain essentially intact (Fig 15a), as bound polymers are generally more stable than freely dissolved polymers in the pore space [34].

In contrast, MX80/PAA^-^ shows only limited polymer degradation according to chemical analyses (Figs 8, 9). Since rheological measurements (Fig 7) and LOI analysis (Table 1) indicate a likely low-molecular-weight polymer fraction in this system, individual chains are unlikely to form multiple bridges between clay particles. Therefore, the proposed model assumes that the cleavage of a single polymer chain into two fragments may suffice to disrupt the initially bridging-dominated structure (Fig 1b). This interpretation is consistent with the observed increase in hydraulic conductivity (Fig 13) and the smectite-dominated viscoelastic response (Fig 14). These macroscopic results indicate a loss of both hydraulic and mechanical reinforcement. Thus, a model is suggested in which polymer-modified connectivity cannot be sustained, leading to a bridge-free and increasingly dispersed fabric (Fig 15b).

Overall, the results demonstrate that polymer–clay systems relying on interparticle bridging are markedly more sensitive to polymer degradation than surface-bound systems. While intact polymer bridges may temporarily persist in stretched configurations [16], their failure leads to a loss of microstructural stiffness and, according to the model, to a microstructural fragmentation. Surface-bound systems, by contrast, exhibit high robustness, as the schematic model suggests that chain scission has only a minor effect on fabric integrity.

### Boundary conditions and experimental design

The applied boundary conditions represent a deliberately simplified and controlled experimental setup. A low vertical stress (10 kPa) and fully saturated conditions with autoclaved inflow water were chosen to reduce the number of influencing factors and to isolate polymer degradation driven primarily by the initial bacterial population present in the bentonite. The solution-intercalation preparation method is well established in the literature, enabling the use of existing data to define and contextualise the initial conditions. Through this procedure, the maximum adsorption capacity of each polymer on the clay surface was adopted as a common reference state. As a consequence, the resulting PAA–bentonite composites had different absolute polymer contents, reflecting the individual adsorption characteristics of the investigated polymers. It should therefore be noted that polymer chemistry (charge-dependent binding) and polymer loading are inherently coupled in the PAA–bentonite systems investigated. Although these conditions do not replicate field situations with variable stress states, unsaturated flow, or site-specific leachates, they are consistent with established approaches aimed at decoupling complex interaction mechanisms. The present study thus provides a mechanistic baseline under well-defined conditions, supporting future investigations under more realistic boundary conditions.

The experimental design prioritised multiple experimental approaches across the hydro-mechanical and microbiological-chemical domains. This allowed polymer–bentonite composites to be characterised from different perspectives, capturing various response variables and behavioural indicators. These results were internally consistent and could be integrated into a conceptual model of polymer–bentonite interactions and degradation processes. However, replication per individual endpoint was limited due to the distribution of permeameter systems across the various analyses. Increased replication in future studies would further strengthen and consolidate these findings.

## Conclusion

This study systematically investigated the biodegradation of polymer-modified bentonite by integrating microbiological, chemical, and rheological evidence. Microbiological analyses enabled the identification of polymer-degrading microorganisms, with an aerobic Pseudomonas species identified as the dominant contributor during the initial degradation phase. A temporal analysis of the evolving soil microbiome demonstrated that polymer degradation was most pronounced within the first approximately six weeks. As the system evolved a shift in microbial community structure toward non-degrading anaerobic bacteria was found due to anaerobic testing conditions, resulting in a pronounced stagnation of further polymer degradation. This temporal degradation behaviour was consistently reflected across all applied chemical and microbiological indicators. While pH and EC measurements provided a first qualitative indication of degradation, the nitrogen balance complemented these measurements by providing mechanistic insights into changes in nitrogen species potentially associated with polymer degradation. The comparative evaluation of different methodological approaches demonstrated that no single method is sufficient for the full characterisation of polymer degradation in bentonite. While chemical and microbiological methods determine polymer loss, hydraulic conductivity measurements and rheological testing enabled the effects of long-term testing on soil fabric to be indirectly determined. By integrating all experimental evidence, a conceptual degradation model was developed. The results clearly indicate that the long-term performance of polymer-modified bentonite is governed not solely by the extent of polymer degradation, but primarily by the initial polymer-clay binding mechanism. Surface-bound polymers (e.g., cationic PAA^+^/MX80) exhibit a degradation-tolerant behaviour, whereas systems dominated by polymer bridging (e.g., anionic PAA^-^/MX80) are inherently susceptible to microscale fabric disruption once degradation initiates. Consequently, a thorough understanding of the initial modification state is essential for interpreting degradation processes and predicting long-term performance. From a civil engineering perspective, these findings are directly relevant to the design and assessment of polymer-modified barrier materials, such as those used in barrier systems. The study demonstrates that polymer degradation can be reliably detected and staged using relatively accessible methods. In addition, the results suggest that bound polymers tend to be more stable than free polymers despite degradation, which is a key factor in the durability of polymer-modified bentonite. Therefore, when selecting and designing polymer-modified bentonite for geotechnical applications, both the polymer-clay binding mechanism and environmental compatibility must be considered in parallel.
